# Short-term photovoltaic energy generation for solar powered high efficiency irrigation systems using LSTM with Spatio-temporal attention mechanism

**DOI:** 10.1038/s41598-024-60672-9

**Published:** 2024-05-02

**Authors:** Muhammad Awais, Rabbia Mahum, Hao Zhang, Wei Zhang, Ahmed Sayed M. Metwally, Jiandong Hu, Ifzan Arshad

**Affiliations:** 1 College of Mechanical and Electrical Engineering, Henan Agricultural University, Zhengzhou, 450002 China; 2 Henan International Joint Laboratory of Laser Technology in Agriculture Sciences, Zhengzhou, 450002 China; 3 State Key Laboratory of Wheat and Maize Crop Science, Zhengzhou, 450002 China; 4 Department of Computer Sciences, University of Engineering and Technology, Taxila, Punjab, Pakistan; 5 Department of Mathematics, College of Science, King Saud University, 11451 Riyadh, Saudi Arabia; 6 Institute for Advanced Study, Shenzhen University, Shenzhen, 518060 Guangdong China; 7 College of Civil and Transportation Engineering, Shenzhen University, Shenzhen, China

**Keywords:** Short-term photovoltaic, Solar power, High-efficiency irrigation systems, LSTM, Spatio-temporal attention, Energy science and technology, Engineering

## Abstract

Solar irrigation systems should become more practical and efficient as technology advances. Automation and AI-based technologies can optimize solar energy use for irrigation while reducing environmental impacts and costs. These innovations have the potential to make agriculture more environmentally friendly and sustainable. Solar irrigation system implementation can be hampered by a lack of technical expertise in installation, operation, and maintenance. It must be technically and economically feasible to be practical and continuous. Due to weather and solar irradiation, photovoltaic power generation is difficult for high-efficiency irrigation systems. As a result, more precise photovoltaic output calculations could improve solar power systems. Customers should benefit from increased power plant versatility and high-quality electricity. As a result, an artificial intelligence-powered automated irrigation power-generation system may improve the existing efficiency. To predict high-efficiency irrigation system power outputs, this study proposed a spatial and temporal attention block-based long-short-term memory (LSTM) model. Using MSE, RMSE, and MAE, the results have been compared to pre-existing ML and a simple LSTM network. Moreover, it has been found that our model outperformed cutting-edge methods. MAPE was improved by 6–7% by increasing Look Back (LB) and Look Forward (LF). Future goals include adapting the technology for wind power production and improving the proposed model to harness customer behavior to improve forecasting accuracy.

## Introduction

The current exorbitant market prices of photon capture devices necessitate the accurate determination of dimensions for photovoltaic (PV) solar power installations prior to conducting any subsequent analysis of their performance in a specific application^1–4^. In order to maximize the profitability of an installation within a limited timeframe, it is imperative to accurately ascertain the power requirements for a given application. Numerous agricultural regions, particularly Char lands, coastal areas, and hilly terrains, lack access to grid electricity, posing a significant challenge for irrigation purposes^5,6^. The utilization of photovoltaic (PV) solar power exhibits considerable potential in various domains, particularly in nations characterized by abundant solar radiation. Notably, the application of PV solar power for water pumping purposes, specifically for irrigating specific crops, emerges as a highly promising avenue^7,8^. The water transportation pumps are equipped with solar cells. The solar energy that is absorbed by the cells is subsequently transformed into electrical energy through the utilization of a generator. This electrical energy is then supplied to an electric motor, which in turn powers the pump^9^. The majority of conventional pump systems primarily operate using either a diesel engine or connecting to the local power grid. Nevertheless, when comparing solar pumps to these two modes of cooperation, certain drawbacks become apparent^10^.

Nonetheless, the inherent instability of the technology remains a significant concern as it hinders the widespread integration of solar power into global power grids^11^. Hence, accurate prediction of solar electricity generation plays a crucial role in enhancing the utilization of solar energy and bolstering the resilience of the power system. The expansion of the solar home system sector has led to the collection of significant time-series data through the use of smart meters. This methodology guarantees the systematic production of precise data, the automated gathering of measurements, and the prompt retrieval of data. The generation of photovoltaic (PV) energy offers numerous advantages to various global markets due to its ability to align peak production with periods of high peak load. Morjaria et al. assert that the cost of solar power production has significantly declined, rendering it highly competitive in various international markets^4,12^. The cost of renewable energy is comparable to or lower than that of power generated from non-renewable sources. Consequently, there has been a notable increase in both the scale and quantity of solar power installations, reaching capacities of several hundred megawatts^13^.

In order to ensure the reliability and effectiveness of power systems, it is imperative to employ robust and reliable models for estimating the power outputs of Photovoltaic systems. This is particularly crucial due to the urgent requirement of integrating environmentally friendly energy sources into grid systems^14^. A limited number of factors exert influence on the overall transitivity of solar irradiance as it traverses the earth's atmosphere. These factors include cloud cover, moisture content, and air pressure^15,16^. The presence of cloud cover has a substantial effect on transitivity, as indicated by previous studies^17,18^. Because of their broad availability, wind and solar photovoltaic energy are increasingly being integrated into electrical components. It is projected that solar energy will account for approximately 11% of power generation, and wind energy will contribute approximately 12% by the year 2050^19,20^. There is a growing focus among governments and individuals in the solar energy sector on the development and utilization of small-scale distributed facilities and self-consumption plants. The user's text does not contain any information to rewrite in an academic manner. The development of accurate and cost-effective forecasting models is crucial to the successful integration of large-scale solar installations and small dispersed systems into electrical infrastructures. However, the unpredictable nature of solar energy poses a challenge to its integration into the grid^22^. Furthermore, there has been a notable rise in the global installed photovoltaic capacity, which has now surpassed nearly 100 GW^23^.

Physical models have demonstrated a high degree of accuracy in predicting weather patterns under consistent conditions. However, their ability to provide enhanced performance in the face of major changes in conditions remains uncertain. The authors of^24^ and^25^ examined a number of commonly used physical models. On the other hand, statistical models are developed based on the mathematical associations between one or more independent variables and particular dependent variables. These findings provide a solid basis for drawing inferences and making projections. The effectiveness of statistical models is greatly influenced by the quality of input data and variations in temporal frames. Among the most well-known and widely-used statistical models are the single exponential model, the dynamically moving average model (ARMA), and its more complex versions (ARIMA, ARMAX, and SARIMA)^26–31^. Machine learning models built from pre-existing statistical models have also been used in various areas of engineering and in scientific study.

The four steps that all these models adhere to are data pre-processing, algorithm training, machine learning model creation, forecast generation, and forecast refinement^32^. ML architectures such as Artificial Neural Networks (ANN)^33^, Extreme Learning Machines (ELM)^34^, and Support Vector Machines (SVM)^35^ are commonly employed for the purpose of estimating power output. Hybrid models mix many architectures for deep learning. Recurrent neural networks (RNNs) and deep learning techniques, which were originally designed for image processing tasks, are just a couple of the many deep learning models that find application across a wide range of domains^36–38^. The projections of photovoltaic electricity using the aforementioned models have shown positive results. Neelesh et al.^39^ proposed a model for optimal onsite solar power generation, and improved the capacity of storage to improve the solar irrigation system. The mechanism was based on several steps such as as data acquisition, soil moisture forecasting, smart irrigation scheduling, and energy management scheme. The suggested system delivered approximately 9.24% water and energy savings for potato cultivation under full irrigation, thereby bolstering the Water-Energy-Food Nexus at the field level. Abhishek et al.^40^ introduced a novel statistical method for forecasting environmental parameters like soil moisture, temperature, and more. Utilizing an Auto-Regressive Integrated Moving Average (ARIMA) model, various hyper-parameter configurations were applied to the local unit's recorded data. The forecasts obtained from the trained ARIMA model undergo validation using four distinct evaluation metrics: Mean Absolute Error (MAE), Mean Squared Error (MSE), Root Mean Squared Error (RMSE), and R2. Results indicated that the model accurately predicts observations with a 99% R2 score. The objective of the research in^41^ was to augment the solar energy collection capacity of Unmanned Aerial Vehicles (UAVs) by integrating solar power to enhance overall energy harvesting systems. The proposed approach merged two distinct renewable systems to harness electricity from the surroundings. Simulation outcomes leveraged an ensemble machine learning algorithm that integrated environmental factors and UAV data to forecast solar power output.

According to reference^42^, this development enabled the extraction of photovoltaic (PV) energy characteristics with enhanced precision. The validation of the PV-Net model's reliability was conducted by employing four performance measures^43^. The authors, in reference to^44^, evaluate the efficacy of photovoltaic (PV) systems by considering the impacts of several parameters. Furthermore, the researchers took into account three separate types of Silicon Photovoltaic (PV) technologies, namely Polycrystalline, Monocrystalline, and Amorphous. Compared to other methods, polycrystalline panels have been shown to be more effective and have a favorable economic impact on power prices. A Convolutional Neural Network-Long Short-Term Memory (CNN-LSTM) design for a photovoltaic (PV) plant with a power capacity of 15 kilowatts (kW) was created in reference^45^. The power generation forecasting model consisted of multiple Long Short-Term Memory (LSTM) layers. The aforementioned observations have served as motivation for previous studies that explore the utilization of hybrid deep-learning architectures in order to forecast the short-term photovoltaic energy output for the subsequent day. The efficacy of the suggested approach was evaluated by contrasting machine learning and deep learning algorithms. The current research suggests a hybrid model for self-consumption photovoltaic installations with the aim of ensuring grid stability, in contrast to previous studies that primarily focused on the development of extensive knowledge and machine learning models for large-scale photovoltaic (PV) plants.

Deep learning models ensure that the right parameters are chosen when used. Nevertheless, the new architecture shows acceptable performance in predicting energy output in terms of accuracy improvements over the original LSTM model. The experimental results show that the suggested model outperforms both deep learning (DL) and machine learning (ML) models in terms of performance.

This paper introduces an attention-based Long Short-Term Memory (LSTM) model that is specifically developed for the purpose of forecasting the power output of a solar plant over various time intervals in the past and future. The dataset used in this study is derived from a photovoltaic facility that is connected to a field irrigation system. It consists of a wide range of variables, such as power generation, climatic factors, and facility power consumption. This study provides a thorough examination of the spatial–temporal attention mechanism, elucidating its visualization and interpretation. This study examines the relationship between the historical data range and the accuracy of forecasting, with the aim of determining the ideal parameters for the predictive model. The effectiveness of the spatial-attention-based LSTM model is assessed through a sequence of tests, demonstrating its favorable influence on the accuracy of forecasting and the robustness of the model. Moreover, a comparison analysis is undertaken to contrast the performance of the proposed attention-based LSTM model with that of different machine learning models, including Decision Trees, K Nearest Neighbors, Support Vector Regression, and Naive Bayes. The evaluation of model performance is conducted with a rigorous approach, employing error measurements such as Root Mean Square Error (RMSE), Mean Absolute Error (MAE), and Mean Absolute Percentage Error (MAPE).

LSTM layers are designed to capture sequential dependencies over time. By incorporating spatiotemporal attention in our proposed system, the model focuses on specific spatial regions at different time steps. This allows the network to dynamically adjust its attention to relevant spatial features as the sequence evolves, capturing intricate temporal patterns. Moreover, this mechanism enhances the network's capability to learn discriminative features, focusing on regions that contribute more significantly to the task at hand.

## Methodology and materials

**Figure 1:**
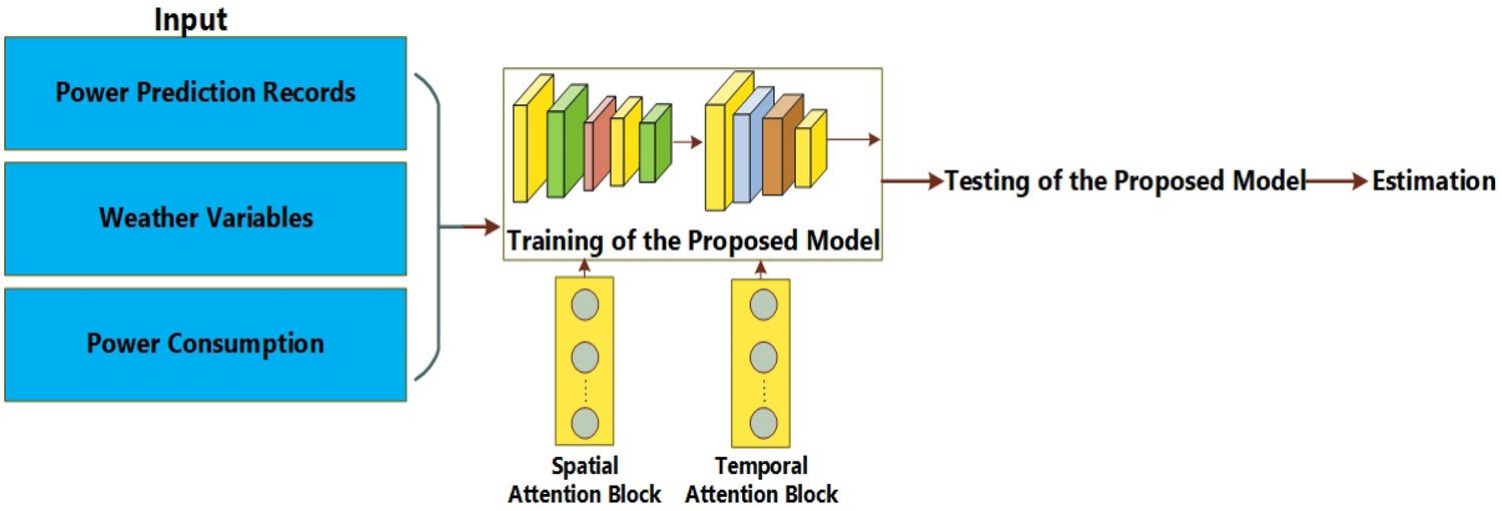
The basic architecture of the proposed system.

This section provides a concise overview of the architecture of the proposed system. The entire process of predicting energy generation relies on multiple stages. Initially, the input undergoes pre-processing by being passed through a pipeline. Subsequently, the pre-processed variables are inputted into the model to undergo feature extraction and classification. In order to extract the most important components from the dataset, an attention mechanism was used in conjunction with the suggested Long Short-Term Memory (LSTM) model. Convolutional neural networks (CNN) and long short-term memory (LSTM) are two deep learning approaches that require organized input throughout the training process^46^. The dataset utilized for the construction of the model is presented in a sequential format and demonstrates a grid topology with one dimension. Figure 1 depicts the diagram of the proposed system.

### Dataset acquisition

This study involved the utilization of a 15 kW photovoltaic (PV) system integrated with a high-efficiency irrigation system. A dataset was collected and analyzed to assess the system's performance. Multiple data aspects were utilized to explore the complexities of a plant's power dynamics. The initial characteristic, denoted as Temperature, represents the midday temperature measured in degrees Celsius. The impact of temperature changes on the operating efficiency of the power production systems within the plant is of significant importance. The second feature, windspeed, measures the mean wind velocity, denoted in kilometers per hour. The significance of this parameter is in its ability to affect the effectiveness of wind-driven power generation methods. Humidity, the third characteristic, is quantified as a percentage and represents the amount of moisture contained in the atmosphere. This particular data point plays a crucial role in assessing the performance of plant operations and the efficiency of electricity generation. The subject of inquiry is the daily allocation of electrical power within a certain grid system. The fourth characteristic is consumption, which is quantified in kilowatts (kW). This statistic offers significant insights into the energy consumption patterns and requirements of the plant. The fifth attribute, Sun Hours, measures the duration of sunny hours that occur on alternate days, represented in hours (h). This characteristic clearly demonstrates the presence of solar irradiation, which has a direct impact on the potential for solar power generation. The sixth characteristic, known as Cloud Cover, is expressed as a numerical value representing the proportion of the sky that is obscured by clouds. This particular characteristic has a substantial impact on the quantity of sunshine that is able to reach the solar panels, hence directly affecting the production of solar power.

**Figure 2:**
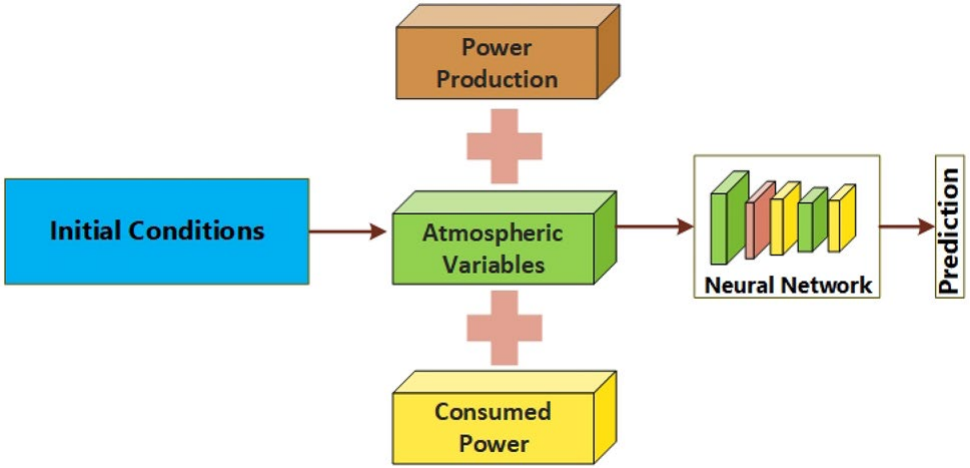
Preparation of dataset.

The ultimate characteristic, production, measures the daily power generation of the facility, denoted in kilowatts (kW). This characteristic is impacted by a convergence of variables such as Temperature, wind velocity, and solar radiation and provides an empirical assessment of the plant's electricity production. By conducting a thorough examination of many data characteristics, this study aims to uncover the complex relationship between weather patterns, energy usage, and electricity generation. The objective is to cultivate a more profound understanding of the plant's overall performance and operational efficiency. There are three distinct kinds of input features to choose from. In the first part of this research project, we investigate the power output records that cover a period of one year and three months. Secondly, weather characteristics such as Temperature, wind speed, and other relevant factors have been obtained by referring to online weather reports. Thirdly, there is a collective comprising the power consumption facility. Additionally, the existing algorithms are given the input variables in order to forecast the power value for the following day (Fig. 2).

### LSTM

**Figure 3:**
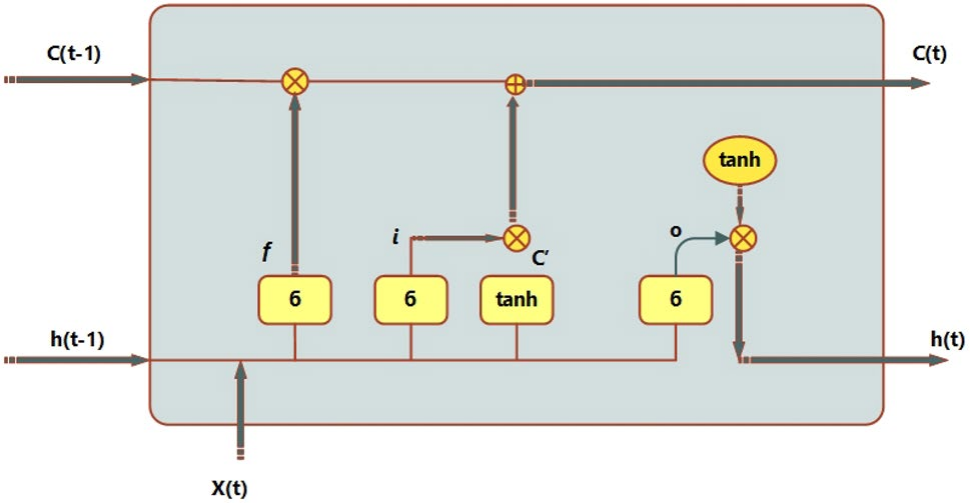
An architecture of LSTM in general.

The normalization layer, RELU layer, LSTM layer, fully connected layer, dropout layer, and classification layer are just a few of the hidden layers that make up the LSTM network. In activities involving sequential data, the long short-term memory (LSTM) model performs better. In this paper, a novel 20-layer Long Short-Term Memory (LSTM) architecture is presented. The design has 21 hidden levels, an output layer, and a feature input layer as its first three layers. The suggested LSTM also includes a mechanism for spatial–temporal attention. A recurrent neural network design called Long Short-Term Memory (LSTM) is capable of accurately capturing the temporal dependencies included in sequential input. The Long Short-Term Memory (LSTM) model uses the prior output of the hidden state, indicated as h(t − 1), and the current input, denoted as c_t, to operate on sequential data^47^. The conventional recurrent neural network (RNN) has problems accurately capturing dependencies, which causes problems like exploding and vanishing gradients to appear, especially when dealing with longer time periods. Long Short-Term Memory (LSTM) models' inclusion of state units, input gates, output gates, and forget gates is a remedy intended to address the vanishing gradient problem, as mentioned in reference^48^. The schematic in Fig. 3 shows how the Long Short-Term Memory (LSTM) model uses input gates, output gates, and forget gates. The input gate, output gate, and forget gate are represented by the sigmoid function, which returns values between 0 and 1, and the hyperbolic tangent function, tanh. Additionally, the state cell memory is represented by Ct, and the candidate for the state of the cell is written as. The forget gate, which is in charge of eliminating unnecessary information from the previous state, as well as the output obtained from the top hidden layer, are among the assigned functions of these gates. The cell's job is to make sure that the output gate chooses the most crucial information, whereas an update gate is in charge of changing the states by adding a new state.

**Figure 4:**
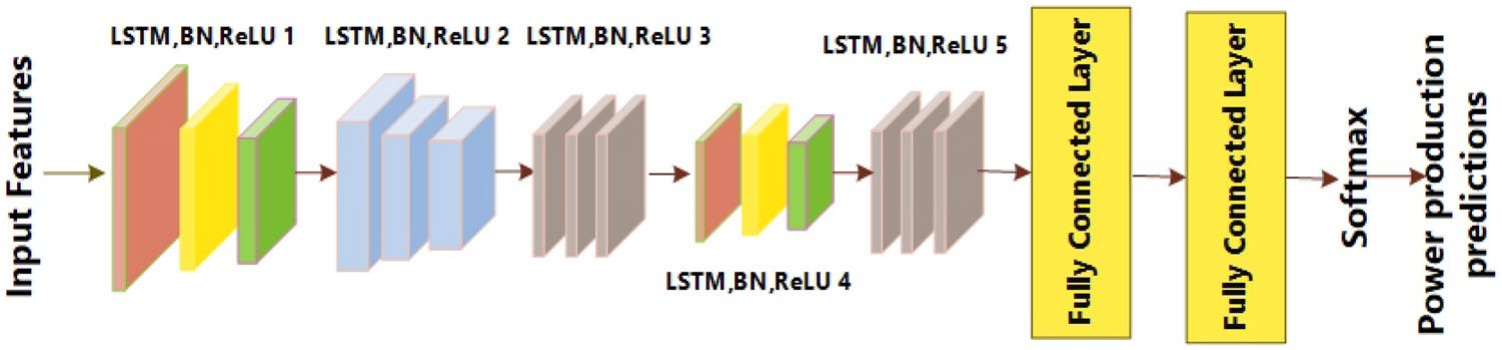
Architecture of basic LSTM-based model.

Long-distance time sequence data can be stored in the LSTM network's short-term and long-term memory. Long short-term memory (LSTM) is used to solve the problem of vanishing gradient, which happens when recurrent neural networks (RNNs) are unable to learn weight parameters successfully. In order to mitigate the problem caused by vanishing gradient, the Long Short-Term Memory (LSTM) architecture makes use of input gates, output gates, and forget gates. Activation functions are provided by the aforementioned gates. The input gate is represented by Eq. (1), the forget gate by Eq. (2), and the output gate by Eq. (3). Equation (4) shows the potential cell state, Eq. (5) shows the memory state of the cell, and Eq. (6) shows the current output. Figure 4 shows the basic architectural layout of the Long Short-Term Memory (LSTM) model.

The feature input layer receives the input features for the network and transmits these characteristics to the hidden layers of the LSTM for data normalization. The inclusion of a batch normalization layer has been shown to enhance the training efficiency of the LSTM network^49^. Batch normalization layers are utilized to execute supplementary scaling and shifting operations. These layers are inserted in order to normalize the input of the activation function and address the issue of vanishing gradient that may occur prior to the hidden layer, such as tanh, RELU, or Sigmoid^50^. The batch normalization layer incorporates two sequential operations, namely normalization and affine transformation, which are applied channel-wise. The normalization operation of data, including n characteristics, includes the calculation of the variance and mean of batch B.

Equation (1) shows the mean, and finally, Eq. (2) is used to calculate the normalization operation by using the mean and variance function.

1 $${\mu }_{B}=\frac{1}{n} {\sum }_{i=1}^{n}{x}_{i}$$

2 $${\sigma }_{B}^{2}= \frac{1}{n} {\sum }_{i=1}^{n}{(x}_{i}-{\mu }_{B})2$$

In this case, B is the mini-batch with input of dimension d, which is the mean and displays variance. The input batch is normalized to have a unit S.D. and a zero mean.

3 $$\widehat{X}=\frac{X-{\mu }_{B}}{\sqrt{{\sigma }_{B}^{2}+ \varepsilon }}$$

Here ${\prime}\varepsilon {\prime}$ is an arbitrary tiny constant utilized to ensure numerical stability before an affine transformation $\widehat{X}$ is used. Equation (4) shows the affine transformation operation.

4 $$\widehat{{\text{y}}\hspace{0.17em}=\Upsilon .X +\upbeta }$$

The learnable scale is ϒ, and the shift parameter is β.

The rectified linear unit (RELU) non-linear function is applied by the RELU layer, a part of neural networks. The rectified linear unit (RELU) is utilized to modulate the output by constraining its range. The utilization of tanh and sigmoid activation functions in the context of backpropagation can give rise to certain challenges. Consequently, we have opted to employ the rectified linear unit (RELU) as an alternative activation function. Equations (5) and (6) provide the mathematical representation of the rectified linear unit (ReLU) in terms of its gradient and function, respectively.

5 $$\frac{d}{dt}ReLU\left(t\right)=\left\{\begin{array}{c}1, t>0\\ 0, t<0\end{array}\right.,$$

6 $$\mathrm{ReLU }({\text{t}}) =\mathrm{max }(0,\mathrm{ t}),$$

If RELU receives a negative input, it returns 0, and if it receives a positive input, it returns the value of t. As a result, the RELU's output ranges from 0 to infinite.

Addition Layer: An addition layer adds inputs element-wise from multiple neural network layers. The addition layer takes multiple inputs of the same shape and returns a single output. We only have to define the number of inputs to the layer when we want to create an addition.

Data is converted into a one-dimensional vector and then given to the fully connected layer, which subsequently processes it. The weights of the connections are multiplied by historical data, and the bias value is applied. Equation (7) illustrates the function carried out by a completely connected layer.

7 $${\text{Fc}}1 =\mathrm{ f }({\text{b}}+{\sum }_{r=1}^{n}{w}_{1},r*{o}_{r}),$$

Here, W stands for weight, b for bias, o for the rth neuron's input vector, and f for the activation function.

Softmax layer: For multiclass classification issues, the softmax layer is employed. Softmax is normally used as an output layer for most multi-classification problems. The function of selection performed by the softmax layer is presented in Eq. (8).

8 $$s\left({v}_{i}\right)=\frac{{e}^{v}i}{{\sum }_{j=1}^{n}{e}^{vj}},$$

The function *s* represents the softmax function applied to the input vector *v.* The variable n denotes the total number of classes. The function *e*^*vj*^ refers to the standard exponential function applied to the input vector, while *e*^*vj*^ represents the standard exponential function applied to the output vector.

Table 1 provides a comprehensive overview of the layers utilized in architecture. Each LSTM layer in the network captures different levels of abstraction in the input sequence. The lower layers learn simple temporal patterns, while the higher layers focus on more complex and abstract representations. This hierarchical feature learning allows the model to understand sequential dependencies at different levels. The use of five concatenated LSTM layers suggests a desire to handle tasks with a high level of complexity. Moreover, the LSTM layers tend to mitigate overfitting by offering a more expressive model capable of capturing intricate patterns in the training data without relying excessively on noise.

**Table 1:** Detail of layers of proposed LSTM network.

Type	Learnable	Activation
Feature input layer	–	7
Long-short-term memory layers × 5	Recurrent weight: 512 × 128	128
	Input weight: 512 × 7	
	Bias 512 × 1	
BN × 5	Scale:128 × 1	128
	Offset:128 × 1	128
ReLU × 5	–	
Fully connected layer 1	Bias:22 × 1	22
	Weight: 22 × 128	
Fully connected layer 2	Bias:22 × 1	22
	Weights:22 × 22	
SOFTMAX	–	22
Class output	–	22

### Proposed attention mechanism

The attention mechanism employed in deep learning approaches operates in a manner analogous to the human visual system, employing attention constraints to select the most salient aspects from a diverse set of input features. Attention blocks have been extensively employed in several applications, such as the detection of brain tumors. Song and colleagues (YEAR) proposed a novel approach for identifying and estimating human actions in films. Their method involves the development of an end-to-end attention block that utilizes spatio-temporal information. Furthermore, Chen et al. (year) proposed a novel approach that incorporates channel and spatial attention mechanisms, as well as an image labeling method, in conjunction with convolutional neural networks (CNNs). This integrated framework demonstrated notable improvements in performance when applied to the specific dataset utilized in their study. Zhai et al. employed a concatenation technique to integrate a dilated convolutional neural network (CNN) with a channel attention model in order to tackle challenges associated with optical flow prediction effectively. The attention-based model utilizing Long Short-Term Memory (LSTM) was created by Ran et al. for the purpose of estimating journey duration. The findings indicate that the utilization of attention-based approaches yields higher levels of accuracy compared to the basic models.

**Figure 5:**
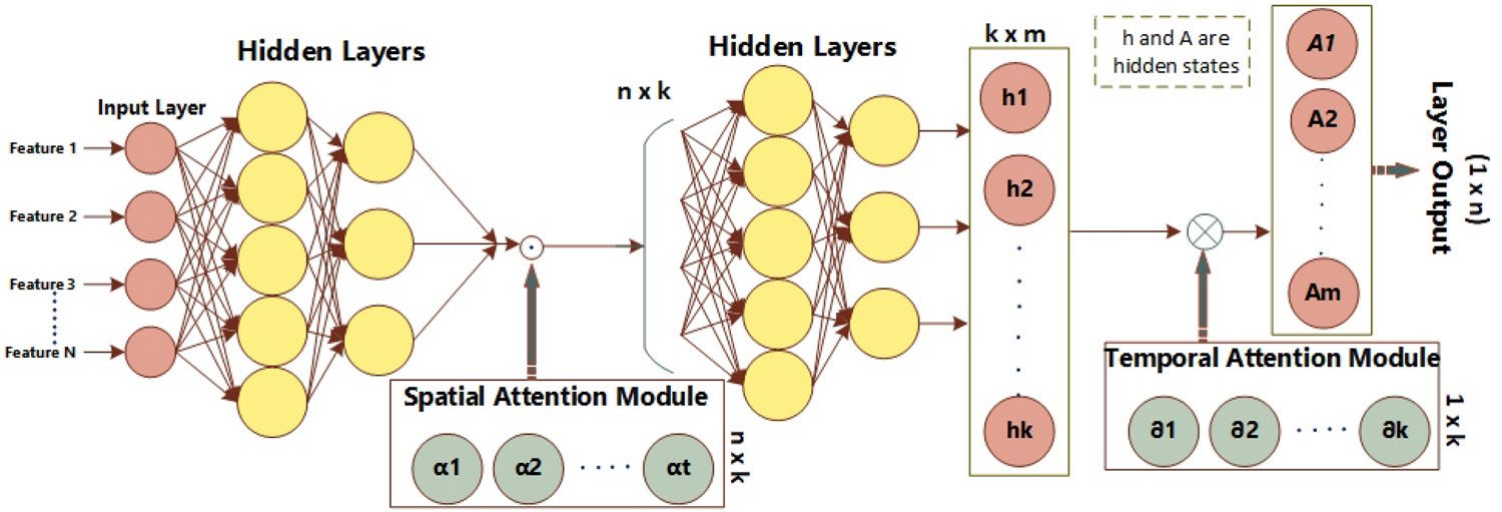
A framework for the proposed spatial–temporal attention-based LSTM network.

The previously described models have been a source of inspiration, prompting us to suggest a spatial–temporal attention mechanism for LSTM. The subsequent part offers a comprehensive examination of the spatial and temporal operation. Figure 5 illustrates the comprehensive architecture of the LSTM model incorporating an attention mechanism.

#### Operation for the spatial attention

Let us consider a spatial–temporal feature vector X, which is a 2-dimensional matrix represented as *XꞓR*^*n*x*k*^. Here, *n* represents the total number of features at a given time step, while *k* is the total number of time steps. The input matrix for the feature can be partitioned into *k n*-dimensional vectors, as denoted by Eq. (9).

9 $${X}_{t}={\{{f}_{1}^{t},{f}_{2}^{t},\dots ..,{f}_{n}^{t}\}}_{n{\text{x}}1},$$

10 $${\alpha }_{t}=SA\left({X}_{t}\right)={\left\{{\alpha }_{1}^{t},{\alpha }_{2}^{t},\dots \dots .,{\alpha }_{n}^{t}\right\}}_{1{\text{x}}n}^{T},$$

11 $${X}_{t}^{\mathrm{^{\prime}}}={\alpha }_{t} . {X}_{t}={\{{\alpha }_{1}^{t}{f}_{1}^{t},{{\alpha }_{2}^{t}f}_{2}^{t},\dots ..,{\alpha }_{n}^{t}{f}_{n}^{t}\}}_{n{\text{x}}1},$$

After the computation of mono-layer neurons, an input vector gets activated using *sigmoid(x)* = *1/ 1* + *e*^*-x*^. ${\alpha }_{t}$ is the spatial weight that is generated by the normalization of *Softmax(x*_*i*_*).* The softmax function is employed to ensure the restricted addition of weights. Moreover, (.) operation presents the Hadamard product, such as element-wise operation.

#### Operation for temporal attention

The data obtained from the spatial attention block is sequentially inputted into the LSTM cell. The data of the hidden layer output is acquired in accordance with Eq. (12).

12 $$H={\{{h}_{1},{h}_{2},\dots ..,{h}_{k}\}}_{k{\text{xm}}},$$

13 $$\partial =TA\left(H\right)={\{{\partial }_{1},{\partial }_{2},\dots \dots ,{\partial }_{k}\}}_{1{\text{x}}k},$$

14 $${H}_{aten}=\partial \mathrm{ x }H= \sum_{i=1}^{k}{\partial }_{i}{H}_{i},{H}_{aten}\in {R}^{1{\text{xm}}},$$

15 $$P=O\left({H}_{aten}\right),P\in {R}^{1{\text{x}}n},$$

**Figure 6:**
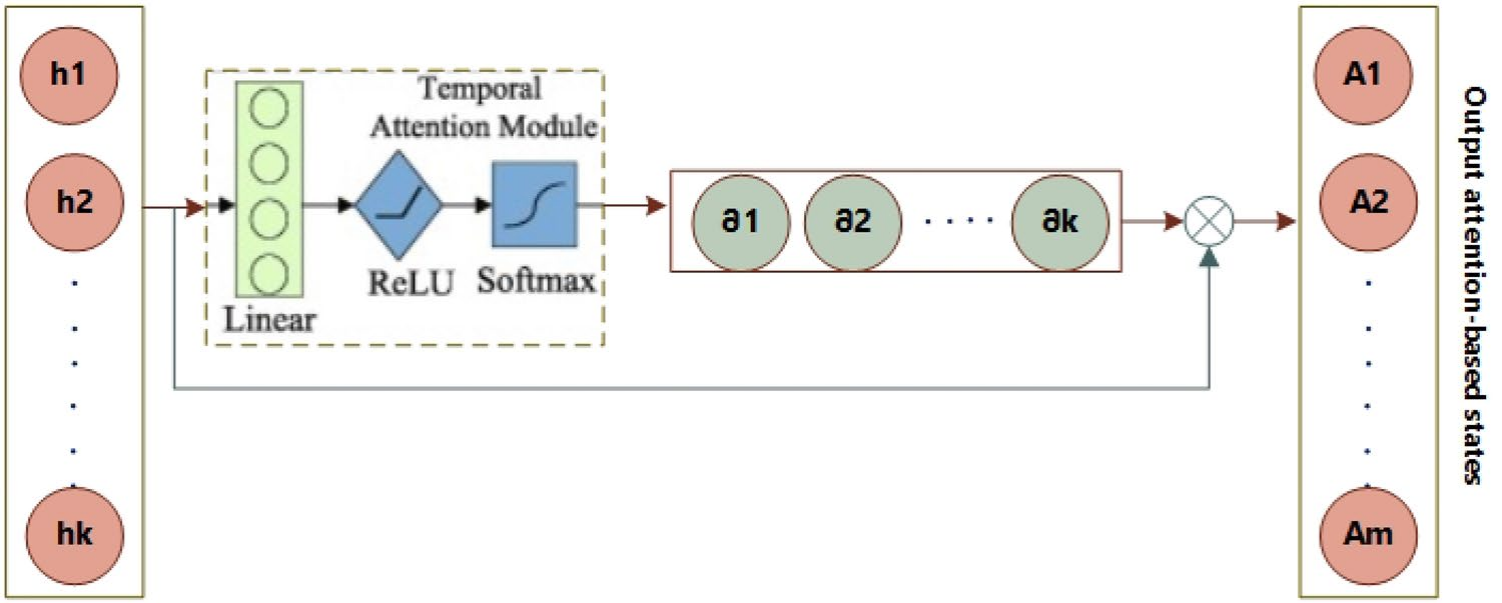
Temporal attention model.

Moreover, the temporal attention bias is established subsequent to the use of the rectified linear unit (ReLU) activation function, denoted as max *(0,x).* Additionally, the process involves the utilization of softmax normalization, as depicted in Fig. 6 and Eq. (19). The variable (*x*) in Eq. (20) denotes the operation of matrix multiplication. Equation (21) presents the final estimation e in the absence of activation.

## Experimental evaluation

In this section, metrics used in the study, experimental setup, and results are presented with details.

### Metrics

In the performance assessment, we included four metrics, namely mean square error (MSE), root mean square error (RMSE), and mean absolute error (MAE). The equations of RMSE, MAE, and MAPE are given below.

16 $$RMSE=\sqrt{\frac{{\sum_{k=1}^{n}({E}_{k}-{E}_{k}^{\mathrm{^{\prime}}})}^{2}}{n}},$$

17 $$MAE=\sum_{k=1}^{n}\frac{|{E}_{k}-{E}_{k}^{\mathrm{^{\prime}}}|}{n},$$

18 $$MAPE=\frac{1}{n}\sum_{k=1}^{n}|\frac{{E}_{k}-{E}_{k}^{\mathrm{^{\prime}}}}{{E}_{k}}|\mathrm{ x }100,$$

Here ${E}_{k}{\prime}$ refers to the estimated energy production, whereas *E*_*k*_ refers to the output for target energy, and *n* exhibits total points of data that are used for the forecasted error estimation. The mean absolute error (MAE) is computed by taking the average of the absolute differences between the target and anticipated values of energy production. In the end, RMSE uses the square to enhance the broad errors.

### Environmental setup

For the execution of the proposed system, we used a single GPU-based system on the Windows 10 operating system. We performed all the experiments using the Python language. The details of the environmental setup are shown in Table 2.

**Table 2:** Environment for the proposed model.

System	Lenovo
RAM	4 GB
Torch	1.3.1
iNLTK library	Default
NLTK library	3.2.6
Pandas	1.4.2
Numpy	1.32.5
Scikit-learn	0.24.1
Keras	5.3.3
Tensorflow	2.4.4

### Machine learning-based models

In addition, we conducted training on various machine learning methods in conjunction with our proposed LSTM network incorporating an attention mechanism. After training the model with suitable values for the number of epochs, batch size, and validation split, a testing phase is carried out to assess the effectiveness of our improved LSTM model. Furthermore, in order to conduct a comparative analysis, we have utilized supervised learning methodologies for the training phase. The classifiers employed for training our machine learning model predominantly consist of decision tree (DT), K nearest neighbor (KNN), support vector regression (SVR), and naïve Bayes (NB).

**Figure 7:**
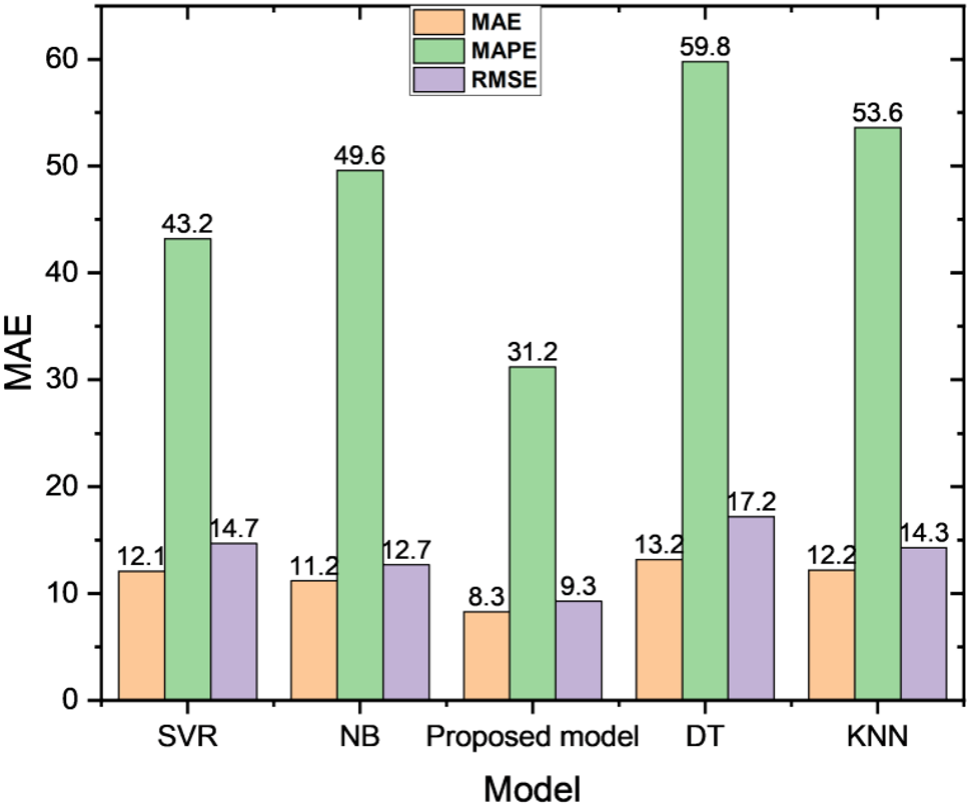
Performance comparison with ML techniques.

**Figure 8:**
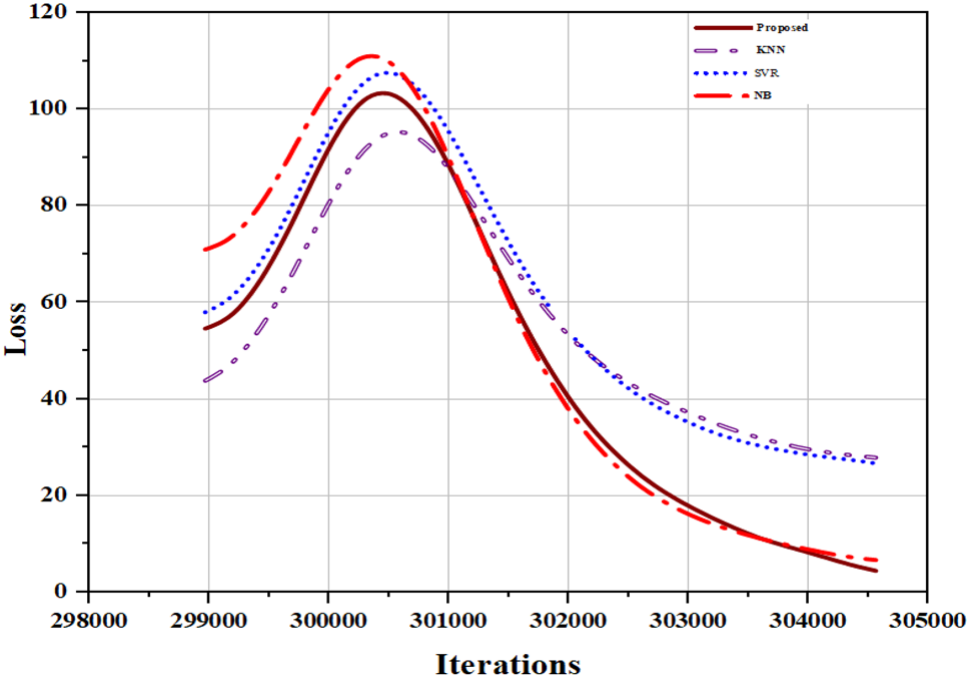
Loss comparison plot among ML algorithms and proposed model.

The performance of these four algorithms is compared with our attention-based LSTM model. The results are reported in Table 3. The comparative analysis plot is shown in Fig. 7. It is clearly exhibited that our proposed model achieves better performance than traditional ML-based techniques. The comparative plot for loss estimation among proposed model and some ML algorithms is shown in Fig. 8.

**Table 3:** Performance over ML-based models.

Model	MAE	MAPE	RMSE
DT	13.2	59.8	17.2
KNN	12.2	53.6	14.3
SVR	12.1	43.2	14.7
NB	11.2	49.6	12.7
Proposed model	8.3	31.2	9.3

### Comparison with base LSTM model

**Figure 9:**
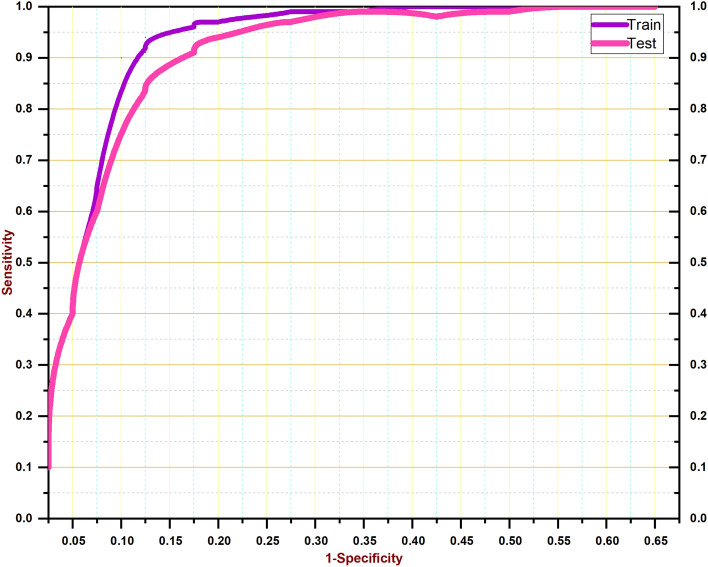
The training and testing curve for the proposed system.

**Figure 10:**
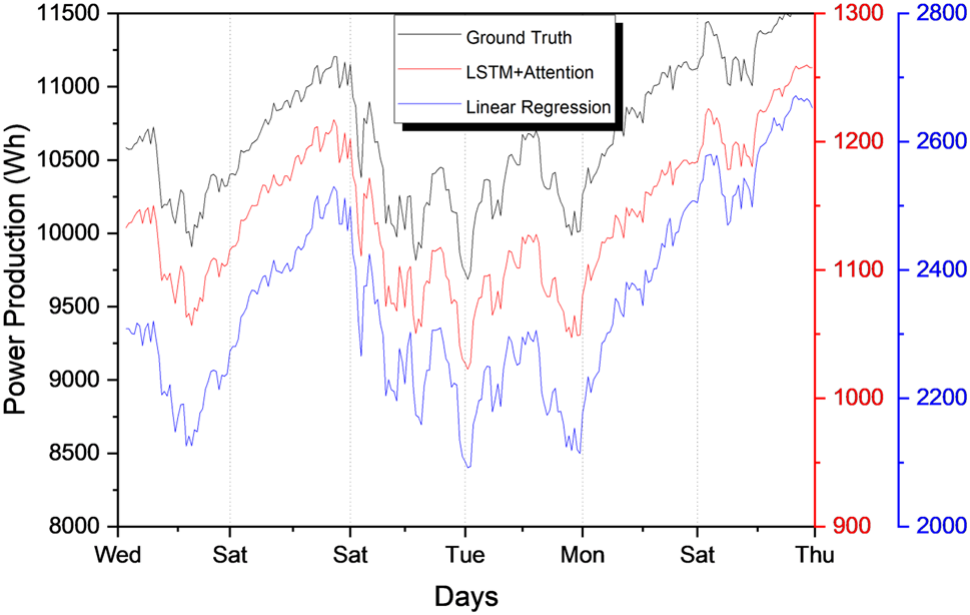
Comparison of our proposed LSTM with Linear Regression and Ground truths.

In this part, we undertake a performance evaluation between our proposed attention-based Long Short-Term Memory (LSTM) model and a baseline LSTM model, taking into account different time strides for both looking back and looking forward. The results indicate that the average mean absolute percentage error (MAPE) of our suggested system is lower than that of the base long short-term memory (LSTM) model, as presented in Table 4. When the number of look-back time steps is raised to 14, the mean absolute percentage error (MAPE) exhibits a decreasing trend in its values. As the duration of observation rises, there is an observable increase in the disparity between the MAPE values of the base LSTM model and our proposed LSTM model. In particular, when examining look back 12, the MAPE of our suggested LSTM model is lower for all look-forward values, including 1, 3, and 7. The observed increase was substantial in magnitude, coinciding with the rise in the Look Forward value. Consequently, we achieved an average improvement of 6–7% in the MAPE. Furthermore, when considering the Look-back values of 14 and 16, we observe the improved performance of the LSTM model that we have provided. The improvements in MAPE are observed to increase when the number of look-back periods is raised up to 16 and when the number of look-forward periods is increased up to 7. Therefore, in all instances, our suggested LSTM model demonstrates superior performance compared to the base LSTM model. The visualization of the training and testing curves is depicted in Fig. 9. The visualization of findings is also depicted in Fig. 10.

**Table 4:** Model performance comparison with base LSTM over various look forward and look back.

Look back	Look forward	MAPE of proposed	MAPE of Base LSTM	MAPE improvements
12	1	19.22	25.137	5.91
	3	20.21	27.24	7.03
	7	23.22	30.34	7.12
14	1	20.22	25.53	5.31
	3	22.33	25.34	3.01
	7	25.31	28.32	3.01
16	1	23.22	29.21	5.99
	3	25.11	33.14	8.03
	7	21.23	29.34	8.11

**Figure 11:**
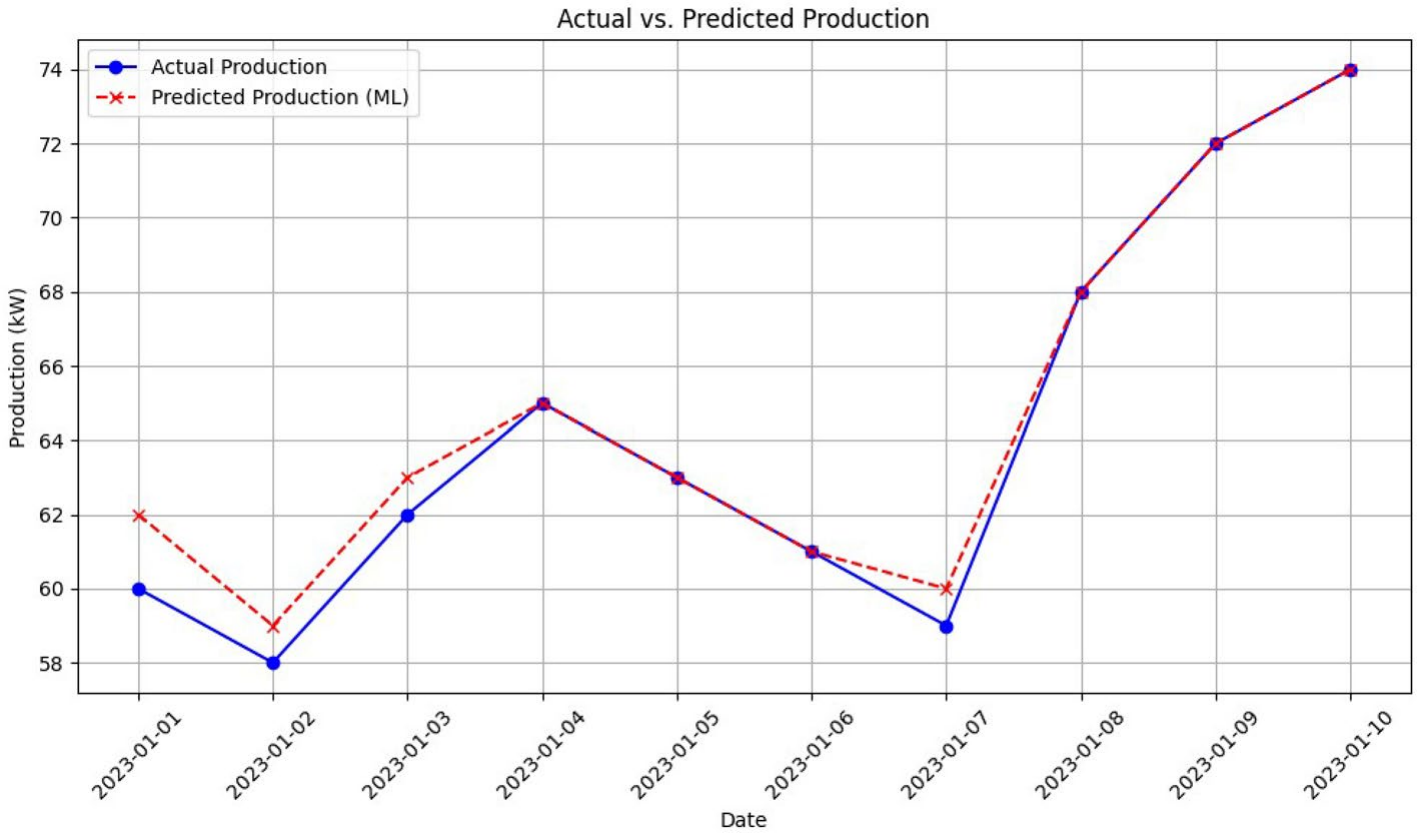
Comparison of our proposed LSTM estimated Production vs Ground truths.

To analyse the performance of the proposed model’s prediction according to the training data, the Fig. 11 is shown. It is clearly notable from the plot that there is tiny difference among the ground truth and predicted values.

### Robustness

Due to the unavailability of public datasets, we used sparse datasets to assess the robustness of the proposed model. Sparse data denotes a dataset characterized by a substantial portion of its elements or features containing zero values.

**Figure 12:**
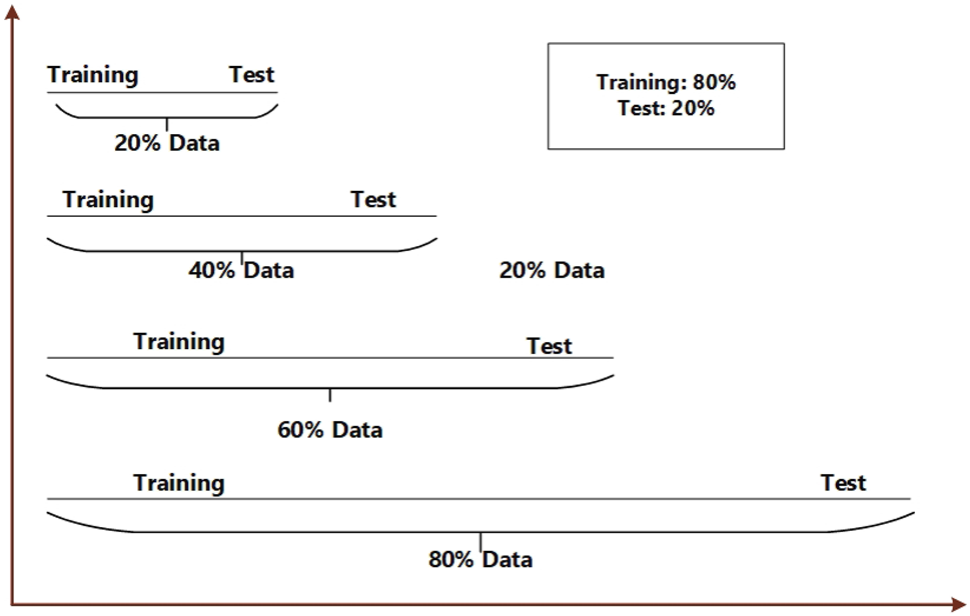
The data-splitting rule for analyzing robustness.

**Figure 13:**
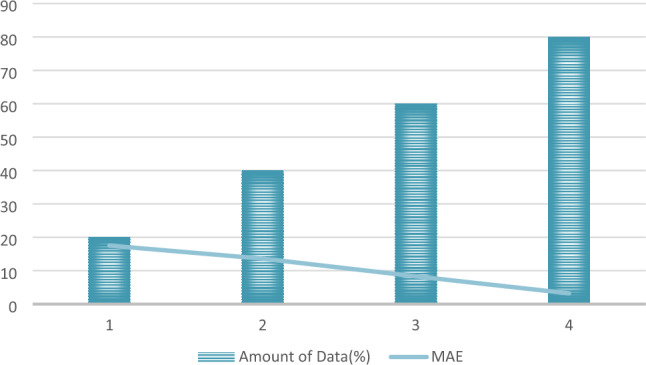
The predicted results by the proposed system on sparse data.

The division of data is illustrated in Fig. 12, considering four distinct scenarios: 20% data, 40% data, 60% data, and 80% data. Figure 13 showcases the predicted outcomes and errors corresponding to varying data proportions. Figure 13 highlights an increase in predicted error as the data amount decreases, underscoring the substantial role of data in model performance. Despite this, the proposed system, incorporating long-short term dependencies and adherence to physical laws, maintains higher accuracy compared to alternative models. This suggests that the proposed model exhibits superior robustness and stability. The findings affirm that the proposed system excels in providing more precise and satisfactory predictions, even in scenarios with sparse data, surpassing the performance of other models.

## Conclusion

In this paper, we propose a robust framework that combines LSTM with a spatio-temporal attention mechanism. Our framework aims to accurately predict the output of a solar plant, specifically in the context of high-efficiency irrigation systems. We accomplish this by carefully selecting and incorporating the most significant meteorological variables that have a direct impact on the power output of the plant. In order to ascertain the effectiveness of the proposed system, a range of machine learning techniques have been utilized and compared against our proposed system. The proposed model has shown significant performance improvements due to the spatio-temporal attention mechanism, which is considered the most valuable information among the hidden layers of LSTM during the training phase. Furthermore, we also took into account the retrospective periods of 12, 14, and 16 days when calculating the MAPE for forecasting periods of 1, 3, and 7 days. We found that the MAPE greatly improved after 14 days. Furthermore, the automated system we propose exhibits flexibility that allows system operators and energy service providers to make optimal decisions about power system control. These decisions encompass various aspects, including demand response, economic dispatch, reservation configuration, and unit effect. However, there exist some challenges that still need attention to overcome, like the unavailability of large data for better network training.

In the future, we aim to utilize this system for wind power production and fine-tune our model to exploit consumer behavioral features to further improve the performance of the forecasting system. Moreover, we will try to cross-validate the performance of the model gathering versatile datasets.

## Data Availability

The datasets used and/or analyzed during the current study are available from the corresponding author upon reasonable request.
