# Author Correction: Short-term photovoltaic energy generation for solar powered high efficiency irrigation systems using LSTM with Spatio-temporal attention mechanism

**DOI:** 10.1038/s41598-024-64983-9

**Published:** 2024-06-26

**Authors:** Muhammad Awais, Rabbia Mahum, Hao Zhang, Wei Zhang, Ahmed Sayed M. Metwally, Jiandong Hu, Ifzan Arshad

**Affiliations:** 1 College of Mechanical and Electrical Engineering, Henan Agricultural University, Zhengzhou, 450002 China; 2 Henan International Joint Laboratory of Laser Technology in Agriculture Sciences, Zhengzhou, 450002 China; 3 State Key Laboratory of Wheat and Maize Crop Science, Zhengzhou, 450002 China; 4 Department of Computer Sciences, University of Engineering and Technology, Taxila, Punjab Pakistan; 5 Department of Mathematics, College of Science, King Saud University, 11451 Riyadh, Saudi Arabia; 6 Institute for Advanced Study, Shenzhen University, Shenzhen, 518060 Guangdong China; 7 College of Civil and Transportation Engineering, Shenzhen University, Shenzhen, China

Correction to: *Scientific Reports* 10.1038/s41598-024-60672-9, published online 02 May 2024

The original version of this Article contained errors.

The name of author Ahmed Sayed M. Metwally was incorrectly given as Ahmed Sayed Mohammed Metwally.

In addition, in the Acknowledgements section,

“This work was funded by the Researchers Supporting Project No. (RSP2023R363), King Saud University, Riyadh, Saudi Arabia. The authors are also thankful to Henan Agriculture University for providing the research facilities.”

now reads:

“This work was funded by the Researchers Supporting Project number (RSP2024R363), King Saud University, Riyadh, Saudi Arabia. The authors are also thankful to Henan Agriculture University for providing the research facilities.”

The original Article has been corrected.

